# A DNA ‘Barcode Blitz’: Rapid Digitization and Sequencing of a Natural History Collection

**DOI:** 10.1371/journal.pone.0068535

**Published:** 2013-07-10

**Authors:** Paul D. N. Hebert, Jeremy R. deWaard, Evgeny V. Zakharov, Sean W. J. Prosser, Jayme E. Sones, Jaclyn T. A. McKeown, Beth Mantle, John La Salle

**Affiliations:** 1 Biodiversity Institute of Ontario, University of Guelph, Guelph, ON, Canada; 2 CSIRO Ecosystems Sciences, Canberra, ACT, Australia; 3 Atlas of Living Australia, CSIRO Ecosystems Sciences, Canberra, ACT, Australia; Fordham University, United States of America

## Abstract

DNA barcoding protocols require the linkage of each sequence record to a voucher specimen that has, whenever possible, been authoritatively identified. Natural history collections would seem an ideal resource for barcode library construction, but they have never seen large-scale analysis because of concerns linked to DNA degradation. The present study examines the strength of this barrier, carrying out a comprehensive analysis of moth and butterfly (Lepidoptera) species in the Australian National Insect Collection. Protocols were developed that enabled tissue samples, specimen data, and images to be assembled rapidly. Using these methods, a five-person team processed 41,650 specimens representing 12,699 species in 14 weeks. Subsequent molecular analysis took about six months, reflecting the need for multiple rounds of PCR as sequence recovery was impacted by age, body size, and collection protocols. Despite these variables and the fact that specimens averaged 30.4 years old, barcode records were obtained from 86% of the species. In fact, one or more barcode compliant sequences (>487 bp) were recovered from virtually all species represented by five or more individuals, even when the youngest was 50 years old. By assembling specimen images, distributional data, and DNA barcode sequences on a web-accessible informatics platform, this study has greatly advanced accessibility to information on thousands of species. Moreover, much of the specimen data became publically accessible within days of its acquisition, while most sequence results saw release within three months. As such, this study reveals the speed with which DNA barcode workflows can mobilize biodiversity data, often providing the first web-accessible information for a species. These results further suggest that existing collections can enable the rapid development of a comprehensive DNA barcode library for the most diverse compartment of terrestrial biodiversity – insects.

## Introduction

Millions of eukaryote species await description, revealing the need for approaches which support accelerated species discovery and description [1]. The billions of specimens in the world’s natural history museums [2] undoubtedly include many new species, but these collections often remain as unsorted accessions, or as undescribed morphospecies. As a consequence, the time between capture, curation, and species description typically spans decades, even for groups under active taxonomic study [3]. Advances in digital imaging and the rise of web-based platforms mean that specimen information can now be mobilized very cost-effectively [4]. However, even if collections possess a workforce dedicated to digitization (and most do not), specimen images and collateral data deriving from new species lack an organizational framework. DNA barcoding provides the opportunity to couple digital specimen data with DNA sequences, enabling the aggregation of specimen records likely to derive from a single species, even those that are undescribed. The framework required to utilize DNA barcodes as an aggregation tool is in place; the Barcode Index Number System provides a persistent registry for DNA barcode clusters, whether they derive from new or known species [5]. Although the capacity of DNA barcoding to accelerate species descriptions has been demonstrated [6], [7], its full potential will only be realized when DNA barcode libraries gain comprehensive coverage for known species.

This study investigates an important question: Can existing specimen collections enable the rapid construction of large-scale DNA barcode libraries or are the barriers created by DNA degradation [8], [9] too serious? This question was addressed through a comprehensive analysis of Lepidoptera in the Australian National Insect Collection (ANIC). Approximately 10,500 species in this order have been described from Australia over the past 235 years, and a similar number are thought to await description [10]. The Lepidoptera collection at ANIC includes more than two million specimens with representatives of about 90% of the known fauna [11]. It also holds thousands of provisional species which have been sorted on morphological grounds, but most lack any near-term prospect of description. As an example, the family Xyloryctidae includes 272 Australian species, the last described in 1964, although there are more than 150 provisional species in ANIC. Without formal documentation, these species-in-waiting contribute nothing towards a deeper understanding of the distributional patterns and diversity of the Australian fauna – information of high importance to both research and conservation.

This project was conducted within the context of the International Barcode of Life Project (iBOL; http://ibol.org) which is assembling DNA barcode records for 100,000 species of Lepidoptera as part of its overall goal of delivering coverage for 500,000 animal and plant species by 2015. The construction of this sequence library for the 5′ segment of the cytochrome *c* oxidase 1 (COI) gene will provide both a platform for automated identifications and a simplified protocol for the validation of provisional species. The efficacy of this approach has gained a comprehensive test in studies on the lepidopteran faunas of Costa Rica [12], Europe [13], and North America [14]. This work has shown that sequence variation in the barcode region discriminates most (>98%) known species and frequently reveals taxa overlooked by prior work [15]. However, past projects have relied heavily on freshly collected specimens to avoid complexities introduced by DNA degradation. This focus has required the activation of expensive field programs and the subsequent identification of newly collected specimens. Given these facts, it would be a key advance if existing collections could be used to build DNA barcode reference libraries.

The feasibility of constructing a comprehensive DNA barcode library for the Australian lepidopteran fauna was examined by analyzing representatives of all known and provisional species in ANIC, except those represented solely by type specimens. This publication reports protocols which accelerated tissue sampling and digitization of these specimens, and enhanced DNA barcode recovery from them. A companion paper will discuss the sequence dataset and consider its implications for the systematics, biogeography, and species diversity of the Australian fauna.

## Materials and Methods

### Specimen Processing

**Figure 1:**
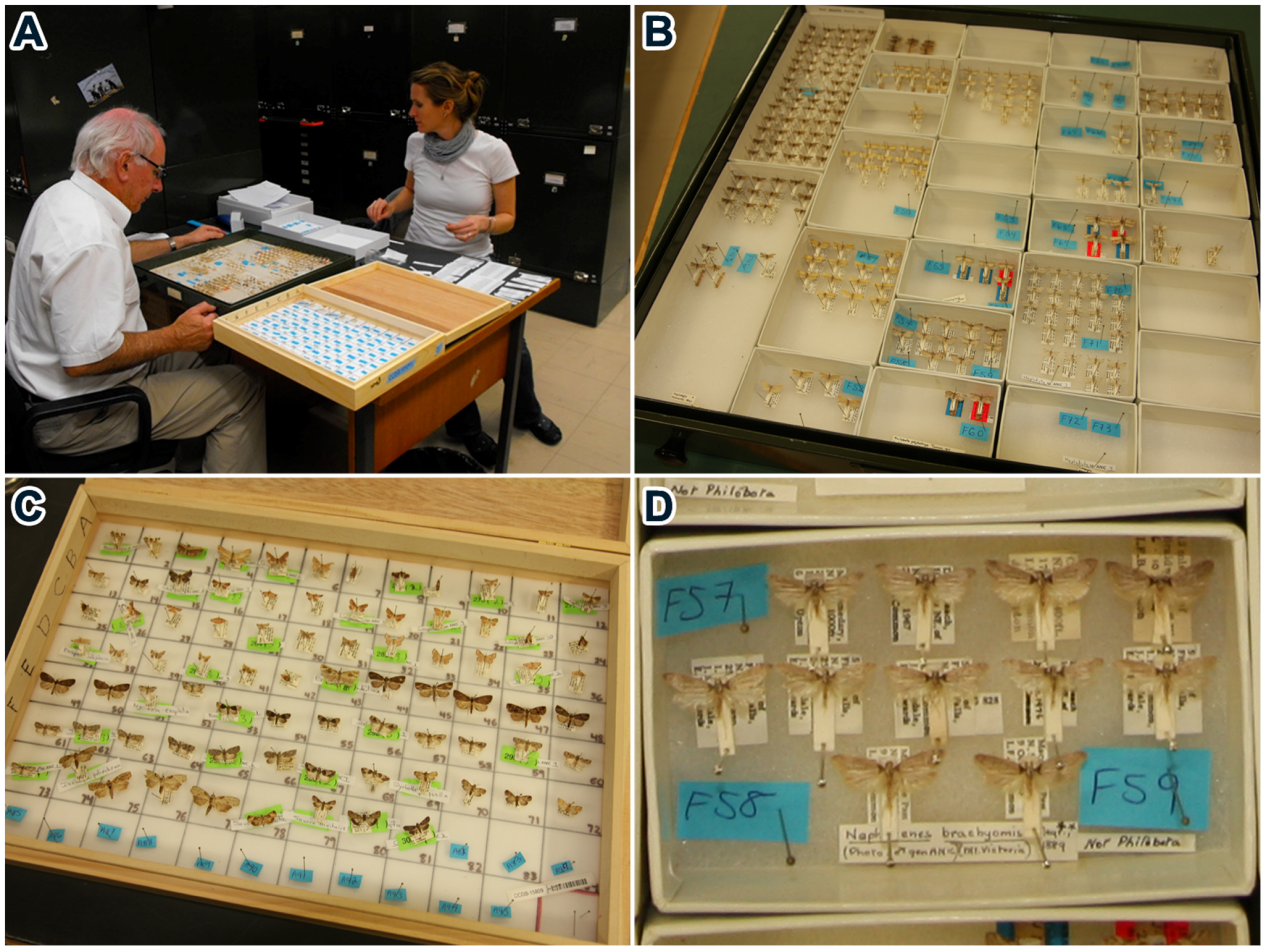
Tracking and arraying specimens. The individuals in this figure have given written informed consent, as outlined in the POS consent form to the publication of their photograph. a) Specimen selection from drawer. b) Drawer with markers in 18 unit trays noting the original positions of specimens selected for analysis. c) Array box partially filled with specimens. d) Unit tray with 11 unanalyzed specimens and three markers for those selected for analysis.

**Figure 2:**
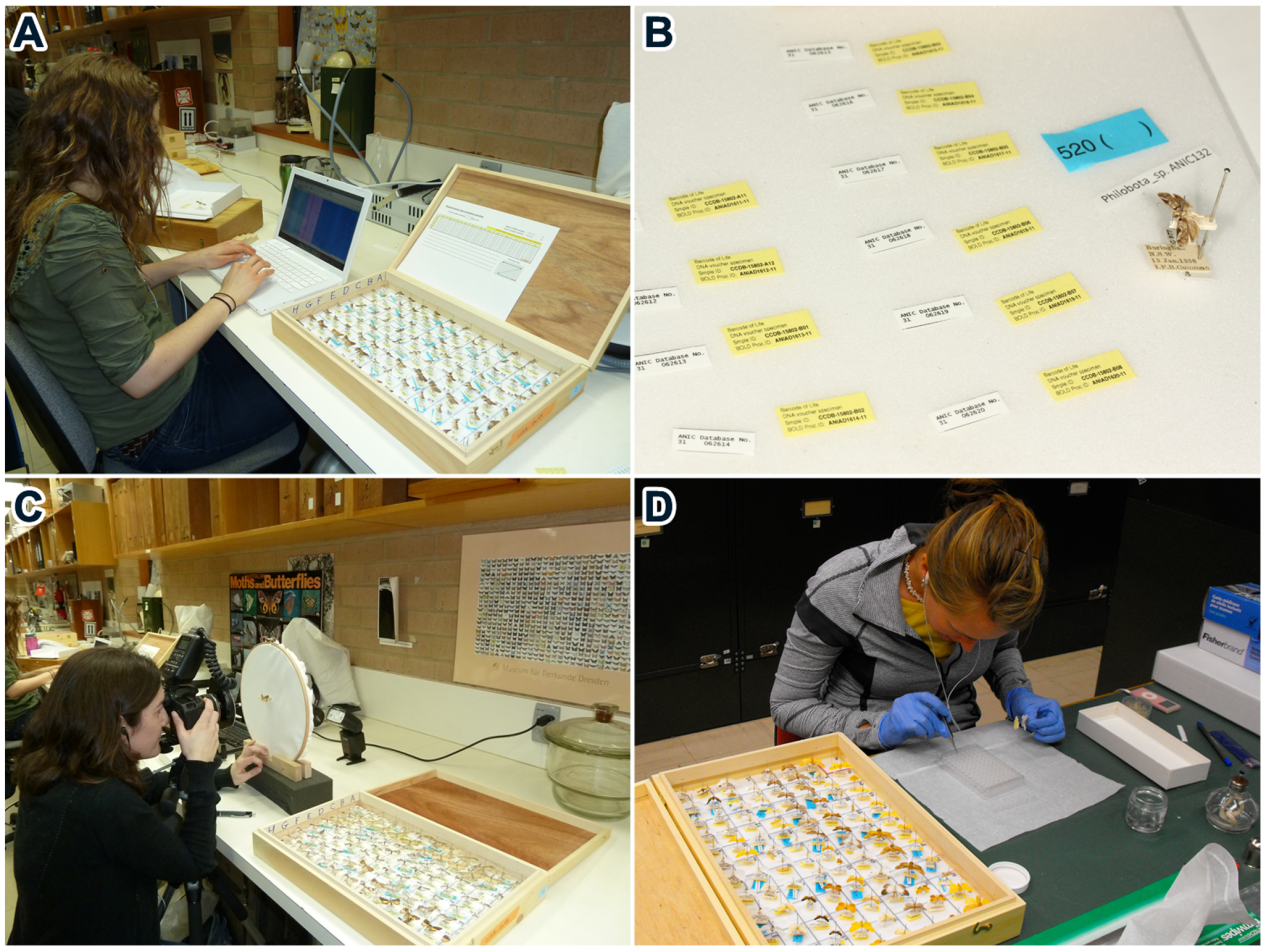
Analytical chain employed to process specimens. The individuals in this figure have given written informed consent, as outlined in the POS consent form to the publication of their photograph. a) Digitization of label information. b) Two labels (DNA barcode, ANIC accession) added to each specimen. c) Photography. d) Tissue sampling.

Permission was granted by the Commonwealth Scientific and Industrial Research Organization (CSIRO) to remove a single leg from specimens of all species of Lepidoptera in ANIC (with the exception of primary types). This permission was granted under a formal Memorandum of Understanding and access to the specimens was provided at no charge. Specimen processing was carried out by five individuals deployed at ANIC for 4–5 weeks on three occasions from October 2010 to December 2011. Members of the team were specialized on particular tasks, but shifted roles when required to aid ‘load-balancing’. Two team members selected specimens, marking the source location of each so that it could be returned after processing to its original position. To aid high-throughput laboratory analysis, specimens were processed in batches of 95 [16], reflecting the inclusion of one blank control in each 96-well DNA extraction plate. To initiate this batch process, specimens were placed in ‘array boxes’ with 96 marked squares and all downstream steps in the workflow focused on these sets of specimens (Figure 1). A third team member digitized information on the existing specimen labels and added a new label linking each specimen to its barcode record (Figure 2). Once the specimen labels were digitized, the array box was passed to a fourth team member, a photographer, who imaged each specimen as well as the entire box to enable quality assurance checks. The box was then passed to the final team member who removed a single leg from each specimen, placing it into a well of a 96-well plate. Each specimen was then returned to its original location. Specimen data and images were uploaded nightly to BOLD (www.boldsystems.org) where they were immediately available to the public. When possible, four to six individuals of each species were processed, seeking broad geographic coverage by selecting a single specimen from each state/territory represented. When multiple specimens were present for a particular region, the youngest was selected. Sampling aimed for comprehensive coverage of all taxa in ANIC, including the many provisional species. Because most of the latter only possessed a generic assignment, and some genera included 10 or more unnamed taxa, all provisional taxa were assigned an interim species name following a standard convention: *GenusA sp*. ANIC01, *GenusA sp*. ANIC02, etc. A total of 12,031 specimens were processed on the first visit, 16,351 specimens on the second, and 13,268 specimens on the final visit.

### DNA Barcode Analysis

**Figure 3:**
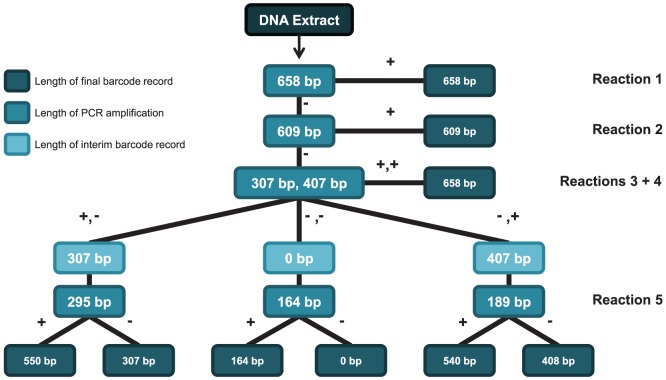
The tiers of PCR employed to recover barcode sequences from Lepidoptera specimens in ANIC.

DNA extracts were prepared at the Canadian Centre for DNA Barcoding using a silica membrane-based method [17], an approach which has shown strong performance in ancient DNA studies [18], [19]. Subsequent analytical efforts involved amplification of the barcode region using multiple PCRs (Figure 3, Table 1). Their large number (134,100) necessitated the use of a Laboratory Information Management System (LIMS) to track the success or failure of particular reactions. All specimens in the first batch were tested for the recovery of four amplicons (658 bp, 609 bp, 407 bp, 307 bp). In cases where none were recovered, a final PCR targeted a 164 bp product. Due to low recovery of the 658 bp and 609 bp amplicons, these rounds of PCR were omitted from the second and third batches. Since barcode compliance requires sequence information for at least 75% of the barcode region (487 bp), an additional PCR was performed on extracts that yielded just a 307 bp or a 407 bp amplicon. For example, those lacking a 407 bp amplicon were assembled into a new plate and tested for the recovery of a 295 bp amplicon which enabled the generation of a 550 bp sequence record when combined with the 307 bp sequence. The LIMS was critical for both the reconfiguration of plates and for the subsequent connection of PCR amplicons and sequences with their source specimen.

**Table 1:** Primer sets employed in amplification of the COI barcode region from Lepidoptera in ANIC.

**Primers**	**Sequence (5′→3′)**	**Amplicon (bp)**	**Reference**
LepF1	ATTCAACCAATCATAAAGATATTGG	658	Hajibabaei *et al*, 2006
LepR1	TAAACTTCTGGATGTCCAAAAAATCA	658	Hajibabaei *et al*, 2006
LepR1	TAAACTTCTGGATGTCCAAAAAATCA	609	Hajibabaei *et al*, 2006
EnhLepF1	CTCCWCCAGCAGGATCAAAA	609	Hajibabaei *et al*, 2006
LepF1	ATTCAACCAATCATAAAGATATTGG	307	This study
MLepR2	GTTCAWCCWGTWCCWGCYCCATTTTC	307	This study
MLepF1	GCTTTCCCACGAATAAATAATA	407	Hajibabaei *et al*, 2006
LepR1	TAAACTTCTGGATGTCCAAAAAATCA	407	Hajibabaei *et al*, 2006
microLepF2_t1	TGTAAAACGACGGCCAGTCATGCWTTTATTATAATTTTYTTTATAG	164	This study
microLepF3_t1	TGTAAAACGACGGCCAGTCATGCWTTTGTAATAATTTTYTTTATAG	164	This study
TypeR1	GGAGGRTAAACWGTTCAWCC	164	This study
TypeR2	GGAGGGTAAACTGTTCAWCC	164	This study
TypeR3	GGTGGATAAACAGTTCAWCC	164	This study
MLepF2_t1	TGTAAAACGACGGCCAGTGCWTTCCCMCGWATAAATAATATAAG	295	This study
microLepR2_t1	CAGGAAACAGCTATGACGTAATWGCWCCWGCTARWACWGG	295	This study
AncientLepF2	ATTRRWRATGATCAARTWTATAAT	189	This study
MLepR2	GTTCAWCCWGTWCCWGCYCCATTTTC	189	This study

Each PCR reaction included 2 µL of DNA, 6.25 µL of 10% D-(+)-trehalose dihydrate (Fluka Analytical), 2 µL of Hyclone ultra-pure water (Thermo Scientific), 1.25 µL of 10× PlatinumTaq buffer (Invitrogen), 0.625 µL of 50 mM MgCl_2_ (Invitrogen), 0.125 µL of each primer, 0.0625 µL of 10 mM dNTP (KAPA Biosystems), and 0.060 µL of 5 U/µL PlatinumTaq DNA Polymerase (Invitrogen) for a total reaction volume of 12.5 µL. Thermal cycling conditions were 94°C for 1 min, 5 cycles at 94°C for 40 s, 45°C for 40 s, 72°C for 1 min, followed by 35 cycles at 94°C for 40 s, 51°C for 40 s, 72°C for 1 min, and a final extension at 72°C for 5 minutes. All amplicons were visualized on a 2% agarose E-gel® 96 pre-cast gel (Invitrogen) and bi-directionally sequenced. Cycle sequencing was performed using a modified BigDye 3.1 Terminator (Applied Biosystems) protocol [20]. Cycle sequencing conditions were 96°C for 1 min followed by 35 cycles at 96°C for 10 s, 55°C for 5 s, 60°C for 2.5 min, and a final extension at 60°C for 5 minutes. Sequencing was performed on ABI 3730×L capillary sequencers (Applied Biosystems), while traces were assembled and edited using CodonCode v. 3.0.1 (CodonCode Corporation).

### Sequence Quality and Data Release

The quality of each sequence was evaluated in two ways – by determining the mean QV score of its trace files and by quantifying the number of uncertain base calls. Sequence records were partitioned into three groups on the basis of the age of their source specimen (1935–1960; 1961–1985; 1986–2010) to facilitate comparison of shifts in sequence quality through time.

Both specimen and sequence data are publically available. Because of the large number of records, they have been released in two data sets. The first (dx.doi.org/10.5883/DS-ANIC1A) assembles 21,746 specimen records (16,925 with sequences) and includes all families from Adelidae to Noctuidae. The second data set (dx.doi.org/10.5883/DS-ANIC1B) includes 19,904 specimen records (14,660 with sequences) for all families from Nolidae to Zygaenidae. The record for each specimen includes a photograph, details on time and locality of collection, a taxonomic assignment, as well as information on PCR protocols. When one or more amplicons were generated, the record also includes trace files, quality scores, sequence information, and a GenBank Accession number.

### Factors Affecting Barcode Recovery

As prior work has revealed that specimen age is an important determinant of sequencing success, the correlation between age and sequence recovery was examined. The impact of variation in body mass among species was also evaluated. This work was initiated by determining dry weights (in µg) for 3817 of the 12,699 species that were sequenced in this study; they included representatives for 1209 of the 2359 genera and for 66 of the 87 families represented. Whenever possible, three individuals of a species were weighed, typically using a Mettler MS balance, but the smallest species (mass <10 ) were weighed on a Mettler XP6U. A mean weight was calculated for the members of each species, each genus, and each family.

The ‘collector-effect’ was examined by analyzing the success in barcode recovery from specimens gathered by seven researchers. Each of these collectors contributed more than 750 of the specimens selected for analysis; their joint efforts accounted for 54% (22,540 of 41,650) of the specimens analyzed. A mass estimate for each specimen was obtained in the following fashion. A direct weight measurement was available for 1771 species which represented 16.7% of the specimens (3772). Another 64.2% of the specimens (14,425) representing 3742 species were assigned the mean weight of other species in their genus, while 18.6% of the specimens (4197) were assigned the mean weight of other species in their family. This estimation strategy left 146 specimens (0.5%) without a weight estimate (because their family lacked representation in the mass registry).

Multiple regression analysis using the IBM SPSS 20 package was employed to examine the relationship between the length of the COI sequence recovered (dependent variable) and three independent variables - specimen age (in years), log body mass (in mg), and collector. Collectors were assigned a categorical value: Common (0), Edwards (1), Upton (2), Nielsen (3), Robinson (4), Keast (5), and Stockard (6).

## Results

### Specimen Sampling

**Figure 4:**
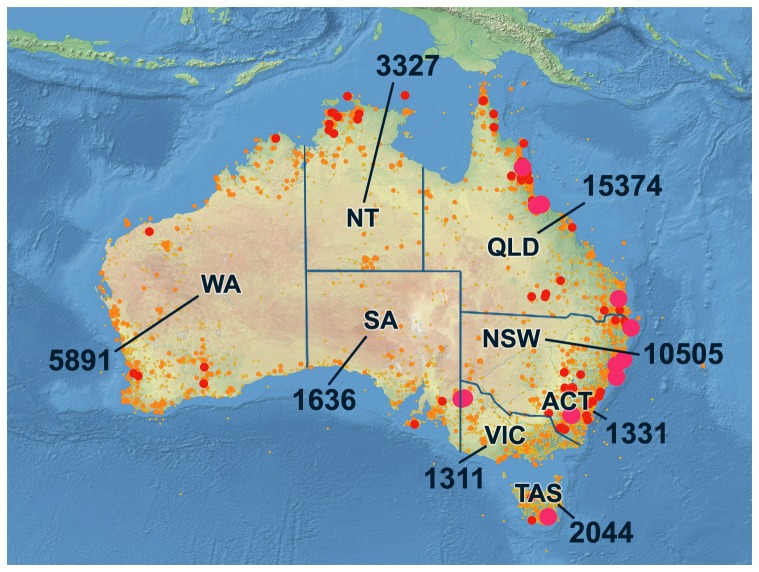
Heat map showing the collection sites for the 41,650 specimens of Lepidoptera analyzed in this study. The numbers indicate the count for each state or territory.

A total of 41,650 specimens were processed over 375 person-days of work, an average of 111 specimens per person-day. These specimens provided coverage for 8245 named species and for 4454 presumptive species for a total of 12,699 taxa. The specimens had an average age of 30.4 years (median = 28.9 years), but ranged from 1 to 112 years. Most derived from Australia, but 216 were from other nations, largely species that were intentionally introduced or that were considered for introduction. Although the specimens provided broad coverage for Australia, sampling effort was not uniform. Nearly 74% of the specimens (30,585) derived from the eastern third (ACT, NSW, QLD, TAS, VIC) of the continent, representing the analysis of 0.011 specimens per km^2^ (Figure 4). By comparison, the number of specimens from the central and western regions was just 0.002 specimens per km^2^.

### Barcode Recovery

**Figure 5:**
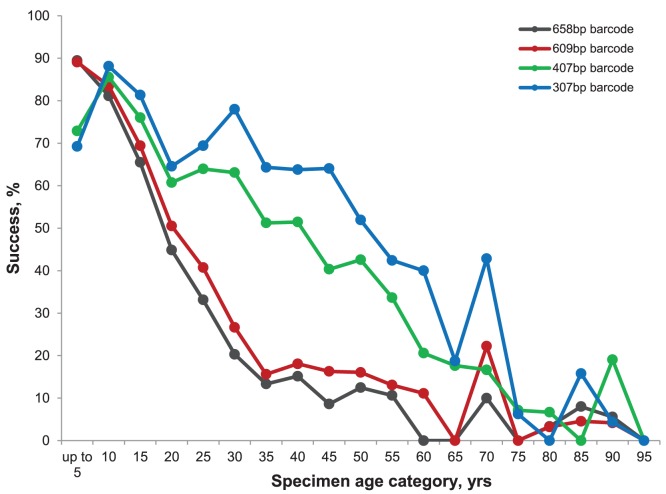
Variation in percentage success in recovery of four COI amplicons from 12,031 Lepidoptera specimens of varied age from ANIC.

**Figure 6:**
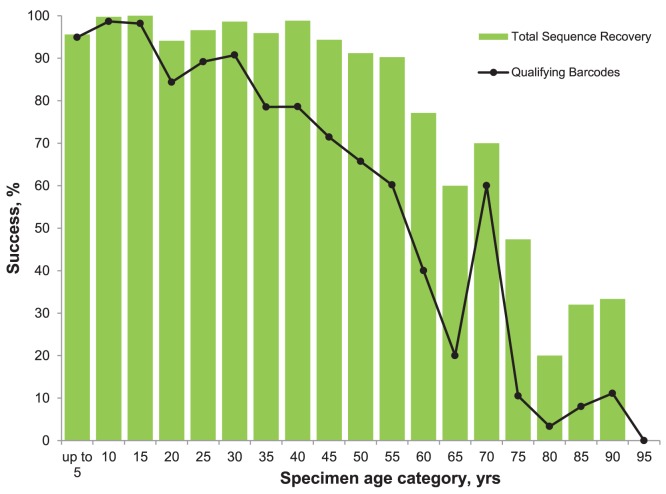
Impact of specimen age on the percentage of specimens yielding either a barcode compliant (≥487 **bp) sequence or a partial barcode sequence.** Results are those for 12,031 specimens of Lepidoptera from the first ANIC batch. These results reflect the overall sequence length after amplification of 307 bp, 407 bp, 609 bp and 658 bp amplicons followed by failure tracking for 164 bp, 189 bp, 259 bp amplicons.

Analysis of the first set of specimens revealed a steep decline in recovery of the 658 bp and 609 bp amplicons in the 30 years following collection from near 100% to 15%. Following this decrease, recovery remained stable for the next 30 years before a further decline to a baseline near 5% (Figure 5). The 307 bp and 407 bp amplicons showed higher success in old specimens with one or both being recovered from more than half of all specimens for 50 years after collection. After the four primary PCRs and subsequent failure tracking, barcode compliant sequences (>487 bp) were recovered from two thirds of specimens up to 55 years old (Figure 6). Although the analytical protocol was simplified for the second and third batches of specimens, a total of 134,140 PCR reactions and 119,997 sequencing reactions were required to complete the analysis. This work led to the recovery of sequences from 31,585 specimens, 75.8% of those analyzed. Barcode compliant sequences (>487 bp) were recovered from 59.2% of the specimens (24,671/41,650) and from 86.1% of the species (10,931/12,699).

There was a clear relationship between the number of specimens analyzed and the recovery of a barcode compliant (>487 bp) sequence for that species (Table 2). In fact, when more than five specimens of a species were analyzed, there was a strong probability of recovering a barcode compliant sequence, even when the youngest was 50 years old.

**Table 2:** Percentage of species with at least one barcode (≥487 bp) record versus most recent collection date following the analysis of varied numbers of specimens.

Specimens	1880s	1890s	1900s	1910s	1920s	1930s	1940s	1950s	1960s	1970s	1980s	1990s	2000s	2010s
1	0	0	0	0	2	3	15	23	37	67	67	80	93	50
2	0	0	0	0	0	2	21	34	51	84	84	90	97	100
3	–	0	0	0	0	0	8	37	59	85	85	91	98	100
4	–	–	–	–	0	0	0	50	82	91	91	94	98	100
5	–	–	–	–	–	–	–	–	75	89	89	96	99	100
6	–	–	–	–	–	–	–	–	100	90	95	97	99	100
7	–	–	–	–	–	–	–	–	–	100	100	97	100	–
8	–	–	–	–	–	–	–	–	–	100	100	96	100	–
9	–	–	–	–	–	–	–	–	–	100	67	100	100	–
>10	–	–	–	–	–	–	–	–	–	–	100	100	99	100

Cells in grey are those where barcode recovery was above 95%. Collection Date (Most Recent Specimen).

### Sequence Quality

**Figure 7:**
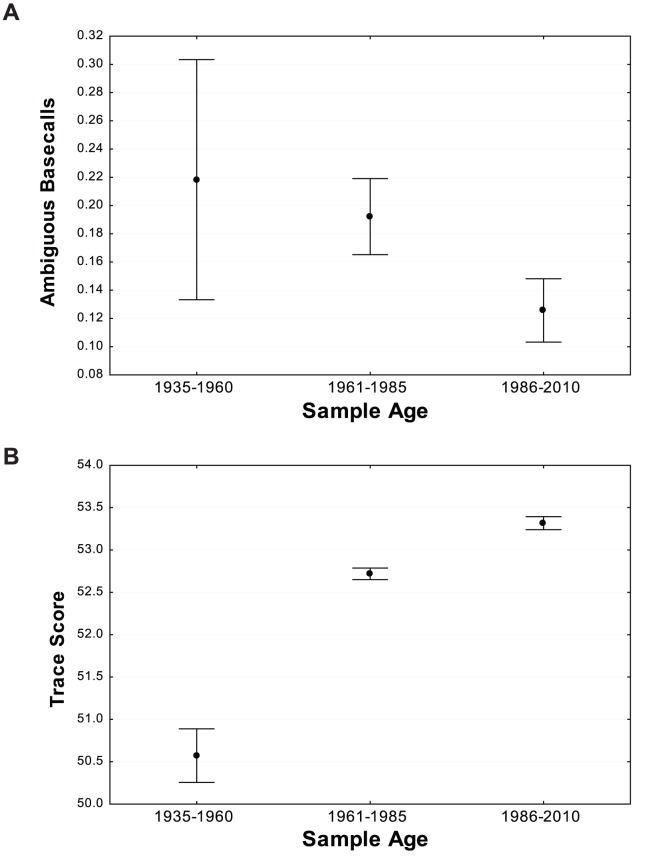
The quality of sequences recovered from ANIC specimens in three age categories as measured by trace scores and by the number of uncertain base calls per kilobase.

**Figure 8:**
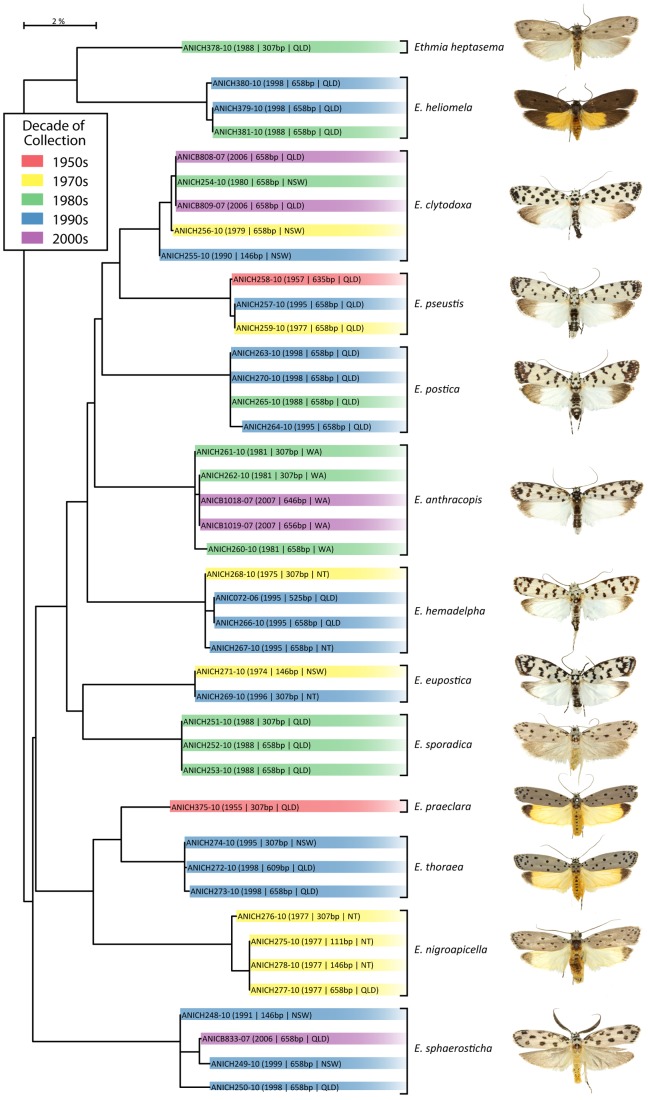
NJ tree (K2P) showing sequence divergence in the barcode region of COI for 42 specimens of *Ethmia* (Elachistidae) collected from 1957 to 2007. Thirteen of the 14 species known from Australia are included.

Trace files were of high quality with an average QV score of 52.9. There was a slight decrease in QV with specimen age (Figure 7a) with scores declining from 53.5 in the youngest category (1986–2010) to 50.5 in the oldest category (1935–1960). The percentage of uncertain base calls was very low, ranging from 0.12 bases per kilobase in the youngest category to 0.24 bases per kilobase in the oldest category. The small impact of specimen age on sequence fidelity was reflected in the observation that specimens of varied age regularly formed tight clusters in NJ trees as shown for species of *Ethmia* collected over a 50 year interval (Figure 8).

### Factors Influencing Barcode Recovery

#### Age effect

**Figure 9:**
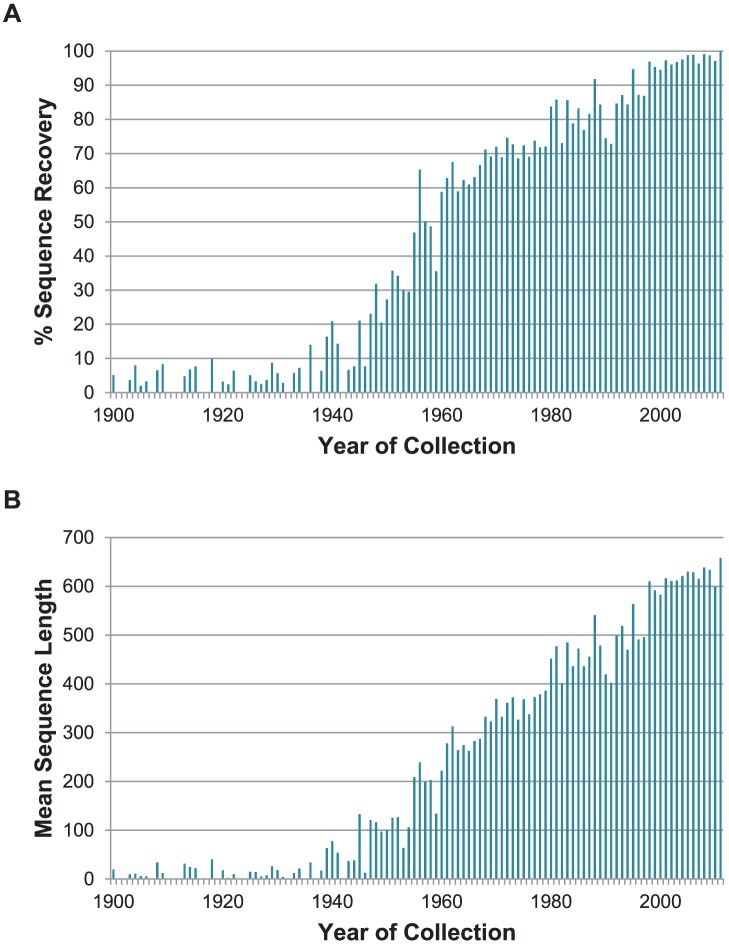
Variation in percentage of specimens yielding a sequence versus collection year and the length of these records for Lepidoptera from ANIC.

Although the fraction of specimens generating a barcode record and the length of sequences decreased with increasing age, nearly 28% of specimens from 50–99 years old yielded at least a partial sequence (Figure 9). However, recovery rates from older specimens were low – just 3% of those more than a century old (11 of 408) delivered any sequence information.

#### Body size effect

**Figure 10:**
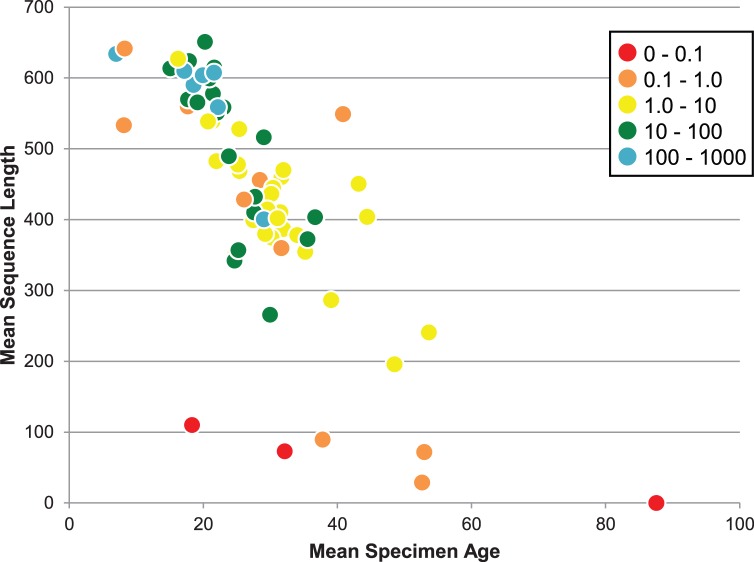
Impact of variation in body size (mg) and specimen age on the mean length of barcode sequences recovered from specimens in 66 families of Lepidoptera sampled from ANIC. Each circle represents a different family while the mean specimen age is the average for all species analyzed in a family. The mean body mass (mg) for species in a family are shown by colouration.

Body size variation had no impact on sequence recovery from specimens analyzed within a decade of their collection as nearly all delivered a barcode compliant sequence. Success in barcode recovery declined through time with the smallest species showing a more rapid decrease (Figure 10).

#### Collector effect

**Figure 11:**
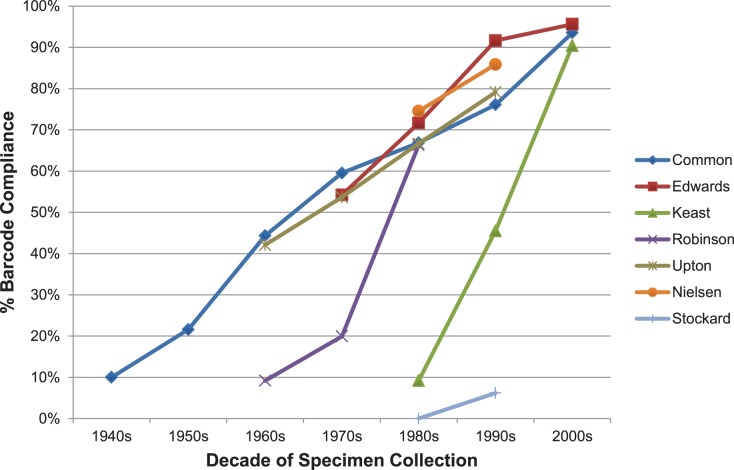
Variation in the recovery of barcode compliant (≥487 bp) sequences from specimens of varying age collected by seven individuals who each contributed more than 750 of the specimens analyzed from ANIC.

The success of barcode recovery varied markedly among specimens gathered by different collectors (Figure 11). Specimens processed by five researchers (Common, Edwards, Nielsen, Upton, Robinson) showed a comparatively slow decline in sequencing success with age, resulting in nearly half of their 50-year old specimens delivering a barcode compliant record. Specimens from Keast showed a different trajectory, one where barcode recovery was initially high, but rapidly decreased. Finally, Stockard’s specimens, although young, were extremely recalcitrant to analysis. Nielsen and Stockard presented a particularly compelling case of divergence. Although their specimens were collected synchronously (1982–1996 versus 1981–1999), and were similar in size (40.7 mg versus 22.8 mg), 80% of the 3030 specimens from Nielsen delivered a barcode compliant sequence, while just 3% of the 794 specimens from Stockard achieved this.

#### Multiple regression analysis

Multiple regression analysis supported conclusions derived from examination of individual factors, as significant effects were evident for age, body mass, and collector (r^2^ = 0.26; Table 3). The overall regression equation indicated that the length of sequence recovered declined by 8.5 bp per year. Body mass also had an important effect on sequence recovery. For example, specimens with a mass of 100 mg produced sequences that were, on average, 166.7 bp longer than those from 1 mg specimens of the same age. Collector effects were also highly significant with specimens collected by Stockard delivering 345.6 bp less than those collected by Common.

**Table 3:** Multiple regression analysis of the impact of specimen age, size, and collector on the length of the sequence recovered.

	R^2^	Coefficient (unstandardized)	Standard Error	P value
*Overall Regression Model*				
Age		−8.51	0.126	<0.001
Body Mass	0.26	83.36	2.566	<0.001
Collector		−52.72	0.963	<0.001
*Regressions By Collector*				
**Common**				
Age	0.20	−8.41	0.223	<0.001
Body Mass		64.04	5.081	<0.001
**Edwards**				
Age	0.18	−8.78	0.282	<0.001
Body Mass		53.22	4.316	<0.001
**Upton**				
Age	0.09	−7.74	0.687	<0.001
Body Mass		95.12	8.030	<0.001
**Nielsen**				
Age	0.10	−4.37	0.895	<0.001
Body Mass		95.49	5.289	<0.001
**Robinson**				
Age	0.14	−6.74	0.473	<0.001
Body Mass		91.47	7.968	<0.001
**Keast**				
Age	0.43	−35.35	1.248	<0.001
Body Mass		125.62	11.508	<0.001
**Stockard**				
Age	0.16	−28.86	3.476	<0.001
Body Mass		95.25	10.266	<0.001

The overall equation was:



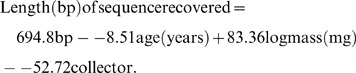



### Synthesis of Specimen Data and Sequences

**Figure 12:**
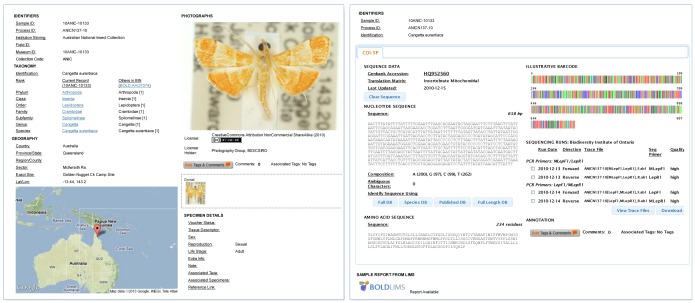
Specimen page and sequence page for one of the 41,650 specimens analyzed from the ANIC Lepidoptera collection.

A barcode record, comprising interlinked specimen and sequence pages, is available on BOLD for each of the 41,650 specimens (Figure 12). The specimen page assembles taxonomic information, an image, GPS coordinates, and other collection details. The sequence page documents the analytical regime including primers, trace files, and the resultant sequence record. As well, each specimen with a sequence record greater than 500 bp has been assigned a Barcode Index Number which indicates its genetic divergence from other taxa, a valuable metric for validating the status of undescribed species.

## Discussion

Prior studies have recovered DNA sequences from museum specimens (e.g. 8, 9), but this project represents the first attempt to analyze representatives of all taxa in a major collection for a hyperdiverse insect group. We found that a five-person team could take 600 specimens per day through all steps of the museum-based phase of the analytical chain. By limiting replication to three individuals per species, 1000 species could be processed in a week. If this productivity were matched in 20 other collections, it would enable one million species to be analyzed in a year. Although there are few collections like ANIC that provide comprehensive, continental-scale coverage for major taxonomic groups, many national and regional collections could contribute in an important way toward the development of a barcode reference library providing broad taxonomic coverage.

While past work has revealed that museum specimens are a challenging analytical target [21], [22], this study has provided new details on factors that impact DNA sequence recovery. Specimen age was confirmed as an important determinant of sequence recovery, but most specimens yielded a barcode compliant sequence if analyzed within 50 years of collection. Moreover, the quality of sequences was high with no evidence of base changes induced by deamination or other sequence artefacts induced by post-mortem base modification. There was a slight reduction in QV scores and a rise in the incidence of uncertain base calls in older specimens, but this reflected the fact that QV scores decreased near the ends (i.e. 20 bp) of each amplicon. Because older specimens typically generated shorter amplicons, a greater proportion of their sequence fell in the terminal regions. A substantial fraction of the final sequence records from older specimens were too short (<487 bp) to qualify for recognition as formal barcodes, but they serve a valuable function, enabling the recognition of full-length sequence records from otherwise unidentified specimens of their species.

Analysis revealed two factors aside from age that impacted sequencing success – specimen size and collection protocols. The body size of species examined in this study varied by six orders of magnitude. When specimens were less than a decade old, these size differences did not impact barcode recovery. However, as age increased, body size became a significant factor with few of the smallest species delivering a barcode compliant record when more than 20 years old. This observation suggests that future work on such groups should either target recently collected specimens or analyze more tissue. The DNA extracts in this study derived from single legs which weighed less than 1 ug for species in small-bodied families. Future work on these groups should aim to extract DNA from abdomens because they weigh 10× more than a leg and likely have higher DNA concentrations because they are tissue-rich. Aside from adjusting analytical protocols to compensate for variation in body size, this study revealed the importance of collection history. Specimens were selected for analysis based on their age and collection site, but sequencing success would have risen if collector had also been considered. The cause of these collector effects is uncertain, but may reflect differential killing agents, treatment during specimen preparation, and/or subsequent storage conditions [22]. The strength of these effects suggests the value of conducting a performance test on a subset of representative specimens from each major collector before launching large-scale efforts at barcode recovery. At ANIC, this analysis would have halted the sampling of specimens from one collector and curbed the selection of older specimens from another. Against this complexity of factors affecting barcode recovery, the analysis of multiple specimens from different localities provided an effective defense although this strategy often cannot be implemented for rare species. The present study focused on developing protocols to process large numbers of specimens at relatively low cost. More elaborate protocols may be appropriate in cases involving the analysis of particularly high value specimens, such as type material or rare species. In such cases, it should, for example, be possible to recover a full barcode sequence by assembling a set of short amplicons [23].

The results from analysis of the ANIC specimens indicate that existing collections of museum specimens, especially insects, can enable the construction of a comprehensive DNA barcode reference library. They also establish that progress can be rapid – a team of five individuals can sample tissues, photograph and digitize data from 10,000 species in three months and a matching team with sequencing expertise can barcode these samples in nine months. Three teams of this size could complete barcode libraries for the Lepidoptera of Australia, Europe, and North America in a year. Coverage for Asia, Africa and South America would require more time, but could still be accomplished in less than a decade. If similar teams were established to lead work on each of the other insect orders, progress would be rapid. We conclude that a comprehensive DNA barcode library for insects is not only feasible, but is almost irresistible.
